# Identification of the best cut-off points and clinical signs specific for early recognition of macrophage activation syndrome in active systemic juvenile idiopathic arthritis

**DOI:** 10.1186/1546-0096-12-S1-P213

**Published:** 2014-09-17

**Authors:** Mikhail M Kostik, Margarita Dubko, Vera Masalova, Ludmila Snegireva, Irina Chikova, Tatyana Kornishina, Eugenia Isupova, Tatyana Likhacheva, Natalia Glebova, Ekaterina Kuchinskaya, Eugenia Balbotkina, Natalia Buchinskaya, Olga Kalashnikova, Vyacheslav Chasnyk

**Affiliations:** 1 Hospital Pediatry, Saint-Petersburg State Pediatric Medical University, Saint-Petersburg, Russian Federation

## Introduction

Macrophage activation syndrome (MAS) – is a severe life-threatening hematological condition, mostly complicated systemic juvenile idiopathic arthritis (SJIA). Early detection of MAS can lead to appropriate therapeutic interventions and change the outcomes. There are no strict criteria for early MAS detection in SJIA. Currently applied HLH criteria can determinate only advanced stage of MAS, which lead to delay diagnosis, late start of specific treatment and associated with poor outcomes. There are several sets of preliminary criteria of MAS in SJIA.

## Objectives

The purpose of our study was to detect early clinical and laboratorial signs able to discriminate macrophage activation syndrome (MAS) from active systemic juvenile idiopathic arthritis (SJIA) without MAS.

## Methods

Our retrospective study was based on reviewing the medical charts of the children, admitted to the rheumatology department with active SJIA and definite MAS (n=18) and without MAS (n=40). We evaluated the data related to SJIA and MAS at the moment of the patient’s admission. If the patient had signs of MAS since admission or developed definite MAS later during this flare he was referred to the main group. The children, who did not have MAS during the flare episode and did not have MAS in the past medical history, were in the control group. We calculated the cut-off points for MAS parameters, performed the analysis of sensitivity and specificity, identified the predictors and provided the preliminary diagnostic rule through “the number of criteria present” approach.

## Results

The clinical signs were relevant to MAS in SJIA: oligoarthicular disease course (OR=5.6), splenomegaly (OR=67.6), hemorrages (OR=33.0), respiratory failure (OR=11.3). The involvement of wrist (OR=0.2), MCP (OR=0.1) and PIP joints (OR=0.1) were protective against MAS development. The best cut-offs for laboratorial parameters were PLT≤211*10^9^/l, WBC≤9.9*10^9^/l, AST>59.7 U/l, LDH>882 U/l, albumin≤2.9 g/dl, ferritin>400 μg/l, fibrinogen≤1.8 g/l, proteinuria. The laboratorial variables were more precise in the discrimination of early MAS than clinical: any 3 or more laboratorial criteria provided the highest specificity (1.0) and sensitivity (1.0) and OR – 2997.

## Conclusion

We detected clinical and laboratorial markers and created preliminary diagnostic (laboratorial) guidelines for early discrimination of MAS in active SJIA.

## Disclosure of interest

None declared.

